# Epiphora before and after upper eyelid functional blepharoplasty: A retrospective cohort study

**DOI:** 10.1371/journal.pone.0255988

**Published:** 2021-08-12

**Authors:** Vannakorn Pruksakorn, Sunee Chansangpetch

**Affiliations:** Department of Ophthalmology, Faculty of Medicine, Chulalongkorn University and King Chulalongkorn Memorial Hospital, Thai Red Cross Society, Bangkok, Thailand; Cairo University Kasr Alainy Faculty of Medicine, EGYPT

## Abstract

Epiphora and dermatochalasis are common presentations in the ophthalmology clinic. To evaluate the change of epiphora before and after functional blepharoplasty, this retrospective cohort study reviewed 39 medical records of epiphora patients who underwent upper blepharoplasty. Severity of epiphora using MUNK score was collected and compared between before and at 6 months after blepharoplasty. The analysis model was performed to measure tear breakup time (TBUT) and frequency of artificial tears use. Subgroups of subjects before blepharoplasty to short baseline TBUT (≤ 10 seconds) and long TBUT (≥ 10 seconds) were also evaluated for the MUNK score change. From the analysis of 39 patients, the results showed a statistically significant decrease in post blepharoplasty MUNK score compared to the baseline (all P < 0.001). There was no significant difference between baseline and post-operative TBUT (P > 0.05). Twenty patients were in the short TBUT group and 19 in the long TBUT group. The reduction of MUNK score after blepharoplasty in the short TBUT group was not different to the long TBUT group (P = 0.50, 95% CI -0.84 to 0.41). However, in short TBUT group, frequency of artificial tears use after surgery was less than pre-operation. From the study, upper eyelid blepharoplasty might be one technique reducing the bothersome epiphora in dermatochalasis patients.

## Introduction

Epiphora or tearing, causes eye discomfort, visual disturbance, annoyance and embarrassment due to multiple dabbing requirement, has a significant effect on many people [1]. Etiologies are multifactorial. Lacrimal gland hypersecretion, evaporative dry eye or ocular surface disease with reflex tearing, obstruction of lacrimal passage outflow, and tear pumping failure from eyelid malposition can aggravate epiphora [2].

Dermatochalasis, the presence of redundant eyelid skin, is one eyelid malposition from the aging process. Puffy eyelid, lateral hooding with skin contact or covering the lateral canthal area, are commonly found in elderly people. The amount of dermatochalasis and upper eyelid contour are different among individuals which not only has a negative effect on appearance, but also eye function. This includes limitation of vision, especially in the superior and peripheral visual fields, orbital discomfort, and dry eye symptoms [3]. However, only one case series addressing the relationship between epiphora, and dermatochalasis has been described [4].

Upper eyelid blepharoplasty is a common eyelid procedure to correct dermatochalasis. Previous studies have reported that blepharoplasty may alleviate several symptoms such as tension headache and increase field of vision [3]. However, epiphora after blepharoplasty is still questioned. In this study, we aimed to evaluate epiphora before and after upper eyelid functional blepharoplasty.

## Materials and methods

This retrospective cohort study was performed after the Institutional Review Board of Faculty of Medicine, Chulalongkorn University (COA 1265/2019) approved the study. This article adhered to the tenets of the Declaration of Helsinki. The Institutional Review Board waived the requirement for informed consent. The authors requested for permission from hospital authorities to review the electronic medical records and collect the data of patients who had history of epiphora and underwent blepharoplasty due to skin redundancy at the Ophthalmology department from one tertiary hospital between January 2016 and August 2019. All data were de-identification. All epiphora patients had history of lacrimal irrigation testing prior to any procedures. The patients who had been proven partial or complete lacrimal passage obstruction by irrigation test were excluded from the study. Those with history of radiation around orbital area, proptosis, previous eyelid and lacrimal surgery, ocular surgery, cataract surgery within 6 months before and after blepharoplasty, trauma around the eyes, and incomplete data were also excluded. The data were collected only from patients who underwent upper eyelid blepharoplasty by one surgeon (VP) to control the surgical technique.

Data including age, gender, history of diabetes mellitus, incidence of lagophthalmos and upper eyelid eversion at 6-month post-operation, subjective epiphora and tear film breakup time (TBUT), and frequency of artificial tears use before and 6 months after blepharoplasty were collected. Subjective epiphora was recorded as severity score based on 0–4 developed by Munk et al. [5]. The higher the score represents more frequent tearing. Tear film breakup time, the objective test to evaluate evaporative dry eye, was determined by measuring time from complete blinking after instillation of topical fluorescein 0.5% to appearance of the first dry spots on the cornea. The average of right eye and left eye TBUT was used for the analysis. Frequency of artificial tears use were recorded by asking patients of the average times of artificial tears use per day within a 1-week period of each visit. Details of demographic data was shown in Table 1.

**Table 1:** Demographic data.

	All	Short TBUT	Long TBUT
Age (years), mean (SD)	64.8 (7.6)	65.3 (7.0)	64.3 (8.3)
Gender, n (%)			
• Female	34 (87.2)	19 (95.0)	15 (79.0)
• Male	5 (12.8)	1 (5.0)	4 (21.0)
Diabetes mellitus, n (%)	27 (69.2)	15 (75.0)	12 (63.2)
Baseline TBUT (seconds), median (IQR)*	9.7 (4.7 to 11.6)	4.7 (3.3 to 8.2)	11.6 (11.0 to 12.6)

*IQR: interquartile range

### Surgical techniques

**Fig 1:**
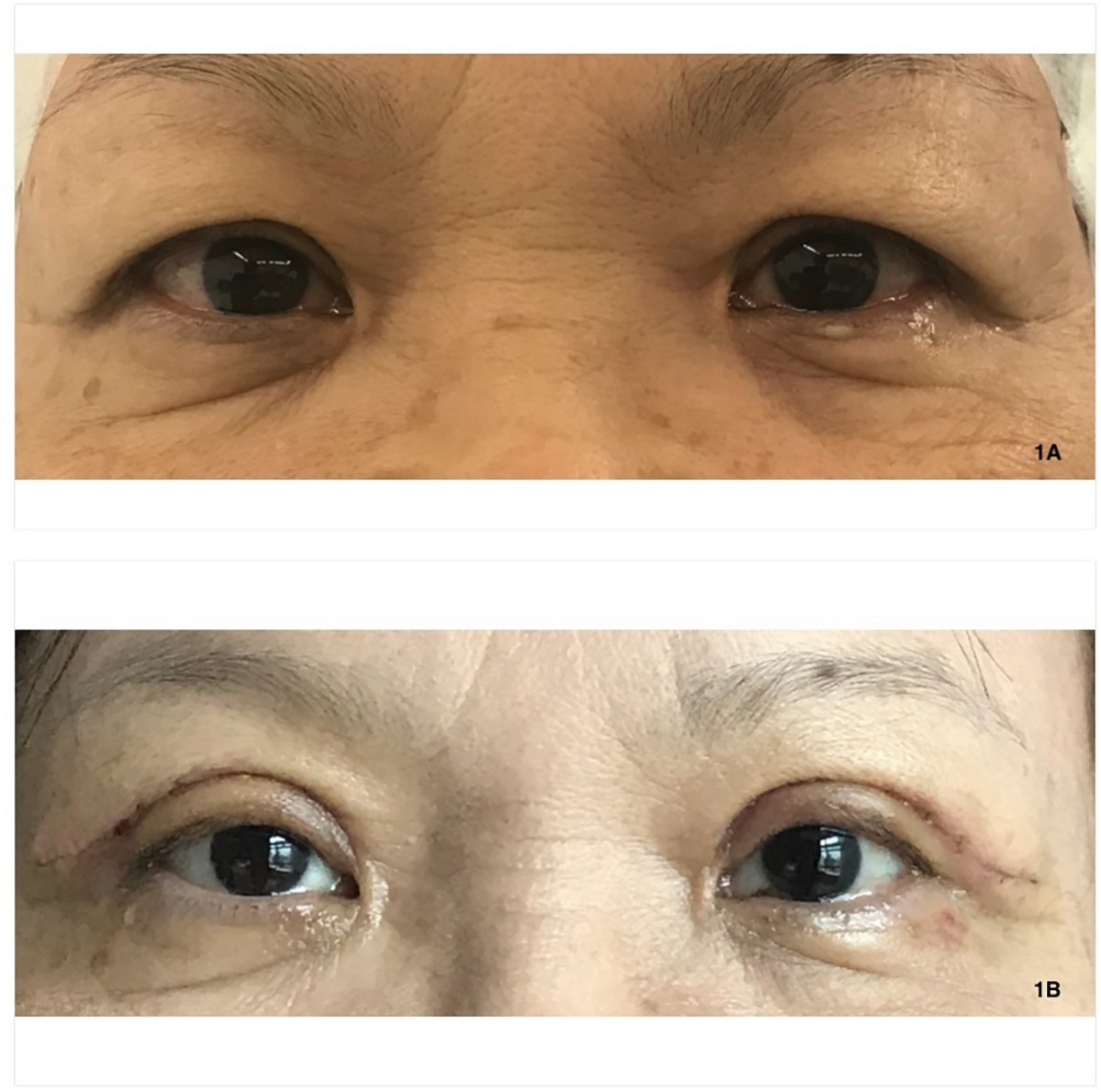
Photo of dermatochalasis patient with lateral hooding. 1A. Before upper eyelid blepharoplasty 1B: One week after upper eyelid blepharoplasty, redundant skin was removed.

Skin marking lines were generated. The inferior margin line was located at the old eyelid crease or 6–7 mm. from the upper eyelid margin. The superior margin line was determined by preoperative pinching of redundant skin. For lateral hooding, the surgeon extended the incisions laterally. The amount of skin removal depended on the severity of skin redundancy of each individual. There must have been enough remaining skin to allow for eyelid closure without eversion and lagophthalmos. Skin excision was done along marking line after local injection of lidocaine 2% with epinephrine 1: 200,000. Partial pre-septal orbicularis oculi muscle was resected only in patients who have redundant orbicularis oculi muscle. Monopolar electrocautery was used for hemostasis. The eyelid crease was formed in patients who had single eyelid crease by fixing thin subcutaneous tissue beneath the skin to peri-tarsal tissue using 6–0 polyglactin-A. Finally, skin was closed in interrupted fashion using 6–0 nylon (Fig 1).

### Statistical analysis

Data analyses were performed using STATA MP 13.0 (StataCorp LP, College Station, TX). Descriptive statistics were calculated. The normality of the data was analyzed using the Shapiro-Wilk test. Measurements at baseline and 6 months post-operation were compared using paired t-test or Wilcoxon signed-rank test, depending on the normality of distribution.

Since MUNK score may be affected by TBUT and artificial tears use, the estimated marginal means (EMM) of MUNK score at each point were calculated along with TBUT, frequency of artificial tears use, and their interactions. The comparison of MUNK score before and 6 months after surgery was then conducted using the EMM with a repeated-measures analysis of covariance (ANCOVA), which included the aforementioned factors as covariates.

Subgroup analysis was conducted to assess whether the changes in MUNK score were comparable between subjects with short and long baseline TBUT. The short TBUT group had preoperative TBUT < 10 seconds. The same analysis models were repeated for the short and long TBUT groups. The absolute change of MUNK score from baseline to 6 months after blepharoplasty were then compared between the 2 groups using t-test. Differences were regarded as statistically significant when the P-value was less than 0.05.

## Results

Data from electronic medical records of 48 patients (96 eyes) were reviewed. The exclusion criteria were identified in 9 patients (18 eyes). Thirty-nine patients (78 eyes) were included in the study. The majority of patients were female (34 patients, 87.18%). The mean age of patients at the time of surgery was 64.76±7.56 years (range: 50–80 years). No patient had history of floppy eyelid syndrome. Baseline and 6-month post blepharoplasty TBUT and frequency of artificial tear use are shown in Table 2.

**Table 2:** Tear breakup time and frequency of artificial tears use.

	All			Short TBUT			Long TBUT		
	Baseline	6-month	p-value	Baseline	6-month	p-value	Baseline	6-month	p-value
Tear breakup time	9.7 (4.7 to 11.6)	9.7 (4.8 to 12.3)	0.15 ^a^	4.7 (3.3 to 8.2)	4.8 (3.7 to 7.0)	0.19 ^a^	11.6 (11.0 to 12.6)	12.1 (11.0 to 13.2)	0.47 ^a^
Frequency of artificial tear use	2 (0 to 8)	2 (0 to 6)	0.001 ^a^	8 (4 to 10)	4 (2 to 8)	0.001 ^a^	0 (0 to 2)	0 (0 to 2)	0.47 ^a^

Data presented in median (interquartile range)

^a^ p-value was obtained from Wilcoxon signed-rank test

There was no significant difference between baseline and post-operative TBUT (P = 0.15). The analysis of MUNK score revealed a statistically significant reduction of postoperative MUNK score from the baseline (all P < 0.001). The analyses are shown in Table 3.

**Table 3:** Comparison of MUNK score at baseline and 6 months post blepharoplasty.

	Baseline	6-month	p-value	95% CI
**Mean of MUNK score (SD)**	**2.95 (0.68)**	**1.10 (0.82)**	**<0.001** ^a^	**1.53 to 2.16**
Short TBUT	3.11 (0.66)	1.37 (0.76)	<0.001 ^a^	1.32 to 2.16
Long TBUT	2.80 (0.70)	0.85 (0.81)	<0.001 ^a^	1.46 to 2.44
**EMM of MUNK score (SE)**	**2.90 (0.15)**	**1.08 (0.14)**	**<0.001** ^b^	**1.87 to 2.19**
Short TBUT	2.93 (0.21)	1.31 (0.27)	<0.001 ^b^	2.03 to 2.44
Long TBUT	2.65 (0.19)	0.90 (0.17)	<0.001 ^b^	1.58 to 2.07

EMM estimated marginal mean; TBUT tear breakup time

^a^ Paired t-test

^b^ Repeated measures ANCOVA

For the subgroup analysis, twenty patients (51.3%) were in the short TBUT group. Age and gender between the short and long TBUT groups were not different (both P > 0.05). Mean MUNK score at baseline was not significantly different between the two groups (P = 0.17). The repeated measures ANCOVA showed a statistically significant decrease in post blepharoplasty MUNK score compared to the baseline for all short TBUT and long TBUT subjects (all P < 0.001).

**Fig 2:**
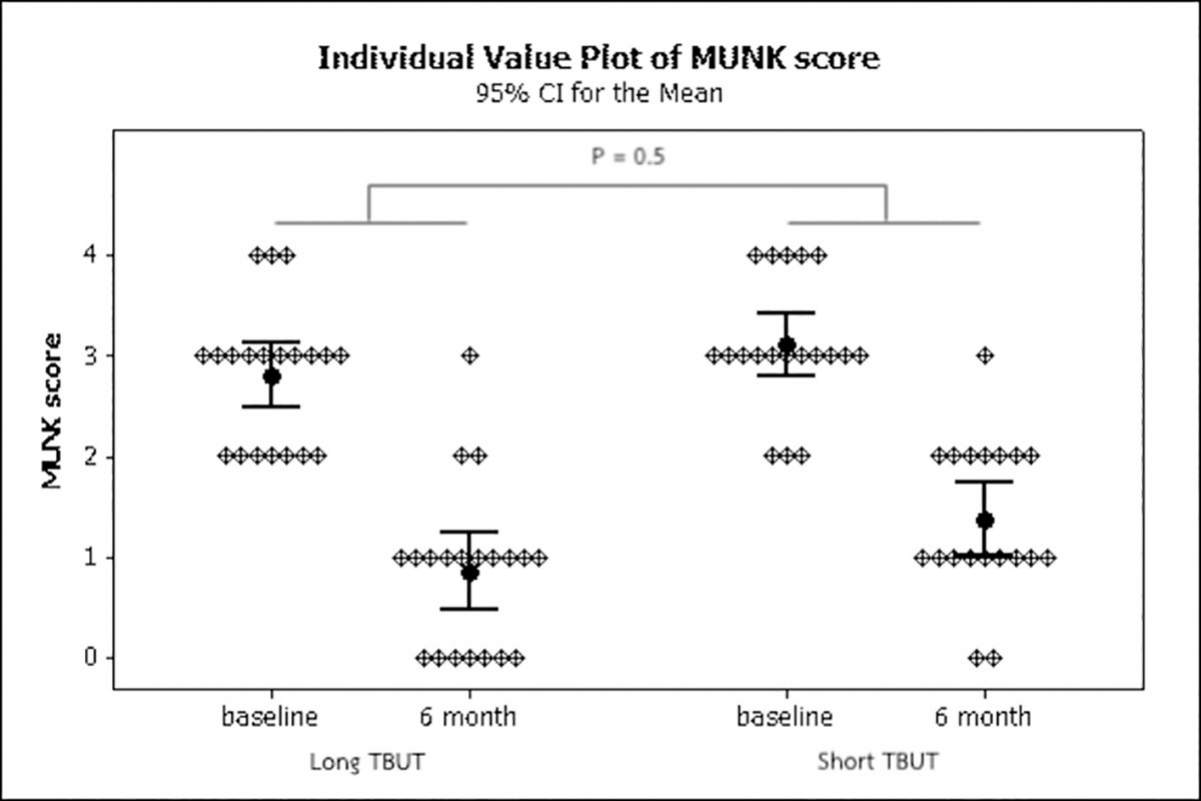
Individual value plot of MUNK score for long tear breakup time and short tear breakup time (black circle dot indicates mean; line indicates 95% confidence interval of the mean).

The reduction of MUNK score from baseline to 6 months after blepharoplasty in the short TBUT group (0.25 ± 0.94) was slightly higher than the long TBUT group (0.16 ± 1.31) but there was no statistically significant difference between the groups (P = 0.50, 95% CI -0.84 to 0.41). (Fig 2) None of the cases developed lagophthalmos or ectropion during the follow-up period.

## Discussion

Epiphora, a subjective symptom of tear overflow onto the face, has been reported to impact many aspects of patient quality of life. Visual function in epiphora patients might be reduced to the same degree as ones awaiting second eye cataract surgery [6]. Shin et al. [1] has reported epiphora can affect many daily activities including outdoor activities, household activities, and interpersonal relationships. Due to multifactorial etiologies [7], management of epiphora is challenging.

Previous studies used MUNK score to evaluate epiphora before and after procedures. Girard et al. [8] used MUNK score to evaluate the results of Botulinum neurotoxin A injection in epiphora patients with patent lacrimal passage and found satisfaction after the procedure. Sipkova et al. [9] also used MUNK score and a questionnaire which was developed for lacrimal surgery to evaluate epiphora. They found a significant improvement in health-related quality of life after surgical correction. However, for eyelid intervention, they focused only on lower eyelid laxity procedures. The current study used MUNK score to evaluate epiphora in dermatochalasis patients who underwent upper eyelid blepharoplasty which has never been evaluated previously. The results demonstrated the significant reduction in epiphora severity after blepharoplasty. The improvement was independent from the TBUT, which is one of the indicators for evaporative dry eye severity. Changing of eyelid contour, eyelash position and decreasing of excessive skin fold, especially around lateral canthal area, might have refined the blink mechanism after the operation and subsequently led to the significant reduction of the MUNK score.

Reflex epiphora has been reported as one presentation of dry eye disease [10]. TBUT is one clinical test for evaporative dry eye. Saadat et al. [11] reported that preserving orbicularis oculi muscle in blepharoplasty can save the tear distribution in patients with dry eye disease. Yan et al. [12] found that dry eye symptoms, especially irritation and TBUT, may increase during the first week after upper eyelid blepharoplasty with partial excision of orbicularis oculi muscle. However, the change recovered within 3 months post operation. In this study, only excessive skin fold and redundant pre-septal orbicularis oculi muscle were resected and postoperative complications including lagophthalmos and upper eyelid eversion which can accelerate tear evaporation were not found. Therefore, TBUT at 6 months after upper eyelid blepharoplasty were not affected. Previous studies have evaluated dry eye condition in blepharoplasty [12–16]. The outcome is still inconclusive. Prischmann et al. reported that 26.5% of patients had dry eye symptoms after blepharoplasty [13]. One study evaluated blepharoplasty patients with subjective dry eye symptoms. The procedure was found effective to relieve symptoms [14]. Other studies evaluated objective dry eye tests including TBUT, Schirmer test, and corneal staining which reported results that were not different from baseline [15, 16]. Similar to the results of this study, postoperative TBUT was not worse than baseline. Therefore, blepharoplasty is likely not to deteriorate dry eye disease. Although the frequency of artificial tears use was subjective evidence, the results showed improvement after blepharoplasty. This might be from less ocular irritation, especially at the lateral canthal area after skin hooding was removed. This was positive for patients not only in relieving epiphora symptoms, but also enhancing their confidence.

It is interesting to note that although blepharoplasty could significantly improve epiphora in both short and long TBUT groups, the 6-month postoperative MUNK score of the short TBUT group was slightly higher. The marginal difference may be an effect of short TBUT to reflex epiphora. However, the difference in the extent of MUNK score reduction between short and long TBUT groups did not reach statistical significance. Skin fold and lateral hooding in dermatochalasis can lead to tear misdirection and collection of tears at the lateral canthal angle, causing epiphora. Blepharoplasty can help with reduction of skin fold and lateral hooding, resulting in a decrease of tear misdirection. This might be the potential cause of the improved epiphora.

There were some limitations in this study. First, this was a retrospective study. Other medications could not be controlled which may impact the results. The frequency of artificial tears use was subjective evidence, which was different among individuals. Patients in this study underwent functional blepharoplasty to correct upper eyelid skin redundancy. These results might not explain patients with cosmetic blepharoplasty. Finally, other objective dry eye tests such as Schirmer’s test and corneal staining and some questionnaires should be used to evaluate and offer more details of dry eye disease in future study.

## Conclusions

Epiphora was significantly improved after upper eyelid functional blepharoplasty. The degree of improvement was similar between subjects with short and long baseline TBUT. Frequency of artificial tears use was also reduced. Upper eyelid blepharoplasty might be one surgical technique to reduce bothersome epiphora, especially in patients who have redundant upper eyelid skin.

## Supporting information

S1 File The raw data. (XLSX)
